# Suggestive evidence of the genetic association of *TMOD1* and *PTCSC2* polymorphisms with thyroid carcinoma in the Chinese Han population

**DOI:** 10.1186/s12902-022-01177-2

**Published:** 2022-10-31

**Authors:** Kaijun Tong, Chang Zhang, Tingting Yang, Rongbiao Guo, Xinyuan Wang, Renyang Guan, Tianbo Jin

**Affiliations:** 1 Department of Medical Images, People’s Hospital of Wanning, Huanshi three eastern Road, Wancheng Town, Wanning City, Hainan Province China; 2 Department of Clinical Laboratory, People’s Hospital of Wanning, Hainan Province Wanning, China; 3 Key Laboratory of Resource Biology and Biotechnology in Western China, Ministry of Education, Northwest University, 710069 Xi’an, Shaanxi China; 4 Provincial Key Laboratory of Biotechnology of Shaanxi Province, Northwest University, 229 North Taibai Road, 710069 Xi’an, Shaanxi China

**Keywords:** Thyroid carcinoma, Single nucleotide polymorphisms, Multifactor dimensionality reduction, *TMOD1*, *PTCSC2*

## Abstract

**Background:**

The purpose of this study was to survey the associations of six single nucleotide polymorphisms (SNPs) in the *TMOD1* and *PTCSC2* genes with thyroid carcinoma (TC).

**Method:**

Peripheral blood samples were obtained from 510 patients with TC and 509 normal controls. Six SNPs were genotyped by the Agena MassARRAY platform. Logistic regression was used to evaluate the association between SNPs and TC susceptibility by calculating odds ratios (ORs) and 95% confidence intervals (CIs). SNP-SNP interactions were analyzed by multifactor dimensionality reduction (MDR).

**Results:**

Our study showed that rs925489 (OR = 1.45, *p* = 0.011) and rs965513 (OR = 1.40, *p* = 0.021) were significantly associated with an increased risk of TC. Rs10982622 decreased TC risk (OR = 0.74, *p* = 0.025). Further stratification analysis showed that rs10982622 reduced the susceptibility to TC in patients aged ≤ 45 years (OR = 0.69, *p* = 0.019) and in females (OR = 0.61, *p* = 0.014). Rs925489 increased TC risk in people aged > 45 years (OR = 1.54, *p* = 0.044) and in males (OR = 2.34, *p* = 0.003). In addition, rs965513 was related to an increased risk of TC in males (OR = 2.14, *p* = 0.007). Additionally, haplotypes in the block (rs925489|rs965513) significantly increased TC risk (*p* < 0.05). The best predictive model for TC was the combination of rs1052270, rs10982622, rs1475545, rs16924016, and rs925489.

**Conclusion:**

*TMOD1* and *PTCSC2* polymorphisms were separately correlated with a remarkable decrease and increase in TC risk based on the analysis.

**Supplementary information:**

The online version contains supplementary material available at 10.1186/s12902-022-01177-2.

## Introduction

Malignant tumors of the thyroid gland account for 1-2% of all malignancies and are the most common endocrine tumors [1]. Thyroid carcinoma has four types, including papillary thyroid carcinoma, follicular thyroid carcinoma, medullary thyroid carcinoma, and anaplastic thyroid carcinoma [2]. The occurrence rate of thyroid carcinoma has rapidly increased worldwide [3]. In the United States, the total occurrence rate of thyroid carcinoma increased 3% annually from 1974 to 2013 [4]. From age 15 to 34, the prevalence increases sharply and then remains stable. Many cases of thyroid carcinoma begin before the age of 30 [5]. The young population of patients with thyroid carcinoma and the increased rate of thyroid carcinoma have attracted extensive attention from social and medical circles. The heritability of thyroid carcinoma (53%) is highest among fifteen common tumors [6]. The causes of thyroid carcinoma are not clearly defined at present. Previous studies reported that thyroid carcinoma was related to the molecular mechanisms of abnormal expression, gene mutation, microRNA, and signaling pathways [7, 8]. A large number of single nucleotide polymorphisms (SNPs), environments, and their interactions cause complex diseases [9]. An increasing number of new risk sites have been identified and validated in different thyroid carcinoma populations [9, 10]. Researchers have discovered some prevalent mutations in thyroid carcinoma, such as *BRAF* V600E and *RAS* mutations [8].

Tropomodulin-1 (*TMOD1*) caps the pointed end of actin filaments and is an important regulator of actin dynamics [11]. Researchers found that *TMOD1* is involved in some cellular processes, such as neurite extension [12], myofibril alignment [13], spine formation, and cell migration [14]. Meanwhile, *TMOD1* has an important role in the regulation of action dynamics in cancer development [15]. Increasing evidence indicates that *TMOD1* is related to several processes of cancer development [11]. For human oral cancer, overexpression of *TMOD1* can intensify regional lymph node metastasis [16]. For cervical cancer, *TMOD1* may directly affect cell motility and cell proliferation as a tumor suppressor [11]. The function of *TMOD1* in thyroid carcinoma is still unknown, although we have known its role in several types of cancer. Studies have shown that individual genetic differences caused by SNPs in DNA coding regions can affect the structure and function of proteins, while individual genetic differences caused by SNPs in noncoding regions can affect gene expression [17]. In addition, genetic polymorphisms in *TMOD1* were significantly associated with human tumors such as oesophageal adenocarcinoma and intracranial aneurysm [18, 19]. Taken together, we propose that *TMOD1* polymorphisms may play a potential role in thyroid carcinoma. However, no study has focused on the association between *TMOD1* polymorphisms and thyroid carcinoma.

Papillary thyroid carcinoma susceptibility candidate 2 (*PTCSC2*), as a thyroid tissue-specific long intergenic noncoding gene [20], includes unsliced and spliced isoforms demonstrating thyroid-specific expression [21]. In European populations, the rs965513 in *PTCSC2* was strongly related to PTC for hypothyroidism, thyroid hormone levels, and other thyroid diseases [22–24]. Age and chronic lymphocyte thyroiditis were related to the unsliced transcript of *PTCSC2* [20]. Rs965513 on *PTCSC2* contributed to increasing the occurrence of follicular thyroid carcinoma and papillary [22]. Rs925489 on *PTCSC2* was correlated with low TSH levels and hypothyroidism [25, 26]. Rs16924016 is an intron variant located in *PTCSC2* and has not reported to be associated with thyroid cancer. Therefore, we wanted to investigate the association of rs16924016, rs925489, and rs965513 on *PTCSC2* with thyroid cancer and their interaction.

The above findings indicate that mutations in the *TMOD1* and *PTCSC2* loci may influence their function in the thyroid gland. Due to ethnic and population differences, this study investigated whether six polymorphic loci of *TMOD1* and *PTCSC2* were related to the susceptibility of thyroid carcinoma to further reveal the genetic pathogenesis of thyroid diseases in the Chinese population.

## Materials and methods

### Characteristics of the study subjects

This study aimed to investigate the association between *TMOD1* and *PTCSC2* variants and thyroid carcinoma in a Chinese Han population. All of 1019 participants (510 cases, 509 controls) were recruited from People’s Hospital of Wanning, Hainan Province, China. The pathological types of 510 patients with thyroid carcinoma included 423 papillary, 49 follicular, 20 medullary, and 9 undifferentiated types. The ethics committee of People’s Hospital of Wanning approved our study. In the study, professional doctors collected demographic information by questionnaires for each subject. All of the subjects signed informed consent forms. The exclusion criteria of the case group included having other cancer history, having metastasized cancers, and without histopathological examinations. The exclusion criteria of the control group included metabolic disease, thyroid diseases, other malignancies, and mental disorders.

### SNPs selection

The detailed steps of *TMOD1* SNP selection were as follows: (1) We obtained the physical position of the *TMOD1* gene on the chromosome 9:97501180–97,601,743 through human e!GRCh37 database (http://asia.ensembl.org/Homo_sapiens/Info/Index). In the VCF to PED Converter window (http://grch37.ensembl.org/Homo_sapiens/Tools/VcftoPed), we entered the gene location, selected the Chinese Han population in the Beijing (CHB) population, and downloaded the ped and info files for the SNPs of *TMOD1*. (2) Then, we used Haploview software for quality control (minor allele frequency (MAF) > 5%, min genotype > 75%, r^2^ < 0.8, and Hardy-Weinberg equilibrium (HWE) > 0.05) to select tag SNPs. Finally, three SNPs (including rs1052270, rs10982622, and rs1475545) were selected for investigation. Three candidate SNPs (rs16924016, rs925489, and rs965513) in the *PTCSC2* gene were selected based on previous literature.

### DNA extraction and SNP genotyping

The study collected 5 ml peripheral venous blood from each subject into an anticoagulation tube containing EDTA-K_2_ and kept them in -80 ℃ refrigerators for DNA extraction and purification. We extracted DNA from venous blood using the whole blood DNA isolation kit produced by GoldMag Co. Ltd., Xi’an in China. A NanoDrop 2000 ultraviolet spectrophotometer (produced by Thermo Fisher Scientific, USA) was used to detect DNA indicators. We designed total primers by the online software of Agena (https://agenacx.com/online-tools/). We genotyped SNPs with the Agena MassArray system of Agena Bioscience from the USA in our study.

### Statistical analysis

We completed all statistical analyses with the SPSS version 25.0 (a software of statistical package for social sciences). The demographic characteristics (age and sex) were tested by χ^2^ test. We checked whether the six candidate genetic loci met HWE. The relevance of different SNPs and thyroid carcinoma was analyzed by calculating the OR and 95% CI using logistic regression. We estimated multiple models (allele model, codominant model, dominant model, recessive model, and additive model) with the wild-type allele as a reference by PLINK software. We adjusted the statistical results by age (based age mean: 45 years old) and sex. The pairwise linkage disequilibrium (LD) was estimated by constructing haplotypes with Haploview (version 4.2). Multifactor dimensionality reduction (MDR) software (version 3.0.2) was used to assess the interaction of candidate SNPs for thyroid carcinoma. All tests were two-sided tests, and *p* < 0.05 was considered statistically significant.

## Results

### Features of subjects

The study enrolled in 510 patients with thyroid carcinoma (361 females and 149 males,) and 509 healthy controls (359 females and 150 males). The mean ages were 45.86 ± 14.71 years old in the case group and 45.91 ± 13.57 years old in the control group. The demographics of the participants included sex, age, tumor type, and lymphatic metastasis status (Table 1). There were no significant distinctions between the case group and control group in sex (*p* = 0.929) or age (*p* = 0.357) (Table 1).

**Table 1:** Demographic characteristic of the cases and controls in thyroid carcinoma

Characteristic		Cases (%)	Controls (%)	***p***
Total		510	509	
Sex	Female	361 (70.8%)	359 (70.5%)	0.929
	Male	149 (29.2%)	150 (29.5%)	
Age (years)	Mean ± SD	45.86 ± 14.71	45.91 ± 13.57	0.357
	> 45	269 (52.7%)	284 (55.8%)	
	≤ 45	241 (47.3%)	225 (44.2%)	
Lymphatic metastasis	Yes	91 (17.8%)		
	No	79 (15.5%)		
	Missing	340 (66.7%)		
Tumor type	Papillary	432 (84.7%)		
	Follicular	49 (9.6%)		
	Medullary	20 (3.9%)		
	Undifferentiated	9 (1.8%)		

SD: Square deviation

*p* < 0.05 indicates statistical significance

### Association between candidate SNPs and thyroid carcinoma risk

Detailed information on the six SNPs is shown in Table 2. All candidate SNPs met HWE (*p* > 0.05). We determined the associations between the SNPs and thyroid carcinoma risk. As presented in Table 3, our results demonstrated that the rs10982622, rs925489, and rs965513 polymorphisms were associated with the susceptibility to thyroid carcinoma. The rs10982622 polymorphism was related to a reduced risk of thyroid carcinoma under the codominant model (OR = 0.74, 95% CI: 0.57–0.96, *p* = 0.025). The rs925489 had an increased risk of thyroid carcinoma under allele (OR = 1.45, 95% CI: 1.09–1.92, *p* = 0.011), codominant (OR = 1.43, 95% CI: 1.05–1.95, *p* = 0.024), dominant (OR = 1.47, 95% CI: 1.08–1.99, *p* = 0.014) and additive model (OR = 1.47, 95% CI: 1.10–1.97, *p* = 0.09). We also found that the rs965513 polymorphism was associated with an increased risk of thyroid carcinoma under allele (OR = 1.40, 95% CI: 1.05–1.86, *p* = 0.021), codominant (OR = 1.37, 95% CI: 1.00-1.87, *p* = 0.048), dominant (OR = 1.41, 95% CI: 1.04–1.92, *p* = 0.028), and additive models (OR = 1.42, 95% CI: 1.06–1.90, *p* = 0.019). Nonetheless, the significant relevance of other SNPs (rs1052270, rs1475545, and rs16924016) and thyroid carcinoma risk were not observed.

**Table 2:** Basic information about SNPs in *TMOD1* and *PTCSC2*

SNP-ID	Chr: Position	Alleles A/B	Gene	CallRate	HWE-***p***	MAF	
						**Case**	**Control**
rs1052270	9: 97,513,142	C/T	*TMOD1*	99.8%	0.368	0.334	0.332
rs10982622	9: 97,542,249	A/G	*TMOD1*	99.7%	1.000	0.327	0.349
rs1475545	9: 97,553,948	C/T	*TMOD1*	99.9%	1.000	0.383	0.378
rs16924016	9: 97,749,049	C/T	*PTCSC2*	100%	0.477	0.176	0.194
rs925489	9: 97,784,318	C/T	*PTCSC2*	99.6%	0.414	0.126	0.091
rs965513	9: 97,793,827	A/G	*PTCSC2*	99.5%	0.414	0.123	0.091

SNP: single nucleotide polymorphism; Chr: chromosome; A/B = minor alleles/major alleles; OR: Odds ratio; 95% CI: 95% confidence interval; MAF: minor allele frequency; HWE: Hardy-Weinberg equilibrium

*p* < 0.05 indicates statistical significance

**Table 3:** The association between *TMOD1* and *PTCSC2* polymorphisms and the risk of thyroid carcinoma

SNP-ID	Model	Genotype	Case (%)	Control (%)	Adjusted by age and sex	
					**OR (95% CI)**	***p***
rs10982622	Codominant	AG	196 (38.4%)	231 (45.7%)	0.74 (0.57–0.96)	0.025
		GG	245 (48.0%)	214 (42.3%))	1.00	
rs925489	Allele	T	891 (87.4%)	918 (90.9%)	1	
		C	129 (12.6%)	92 (9.1%)	1.45 (1.09–1.92)	0.011
	Codominant	CT	117 (22.9%)	88 (17.4%)	1.43 (1.05–1.95)	0.024
		TT	387 (75.9%)	415 (82.2%)	1.00	
	Dominant	CC.CT	123 (24.1%)	90 (17.8%)	1.47 (1.08–1.99)	0.014
		TT	387 (75.9%)	415 (82.2%)	1.00	
	Additive	/	/	/	1.47 (1.10–1.97)	0.009
rs965513	Allele	G	893 (87.7%)	918 (90.9%)	1.00	
		A	125 (12.3%)	92 (9.1%)	1.40 (1.05–1.86)	0.021
	Codominant	AG	113 (22.2%)	88 (17.4%)	1.37 (1.00-1.87)	0.048
		GG	390 (76.6%)	415 (82.2%)	1.00	
	Dominant	AA.AG	119 (23.4%)	90 (17.8%)	1.41 (1.04–1.92)	0.028
		GG	390 (76.6%)	415 (82.2%)	1.00	
	Additive	/	/	/	1.42 (1.06–1.90)	0.019

SNP: single nucleotide polymorphism; OR: odds ratio; 95% CI: 95% confidence interval

*p* < 0.05 indicates statistical significance. /: indicates additive model

### Analysis of stratification based on age and sex

The associations stratified by age and sex are shown in Table 4. After stratifying by age, we found that the rs10982622 polymorphism was associated with a decreased risk of thyroid carcinoma in people aged ≤ 45 years under the codominant model (OR = 0.61, 95% CI: 0.41–0.90, *p* = 0.014). For people aged > 45 years, rs925489 had risk-increasing effects on thyroid carcinoma under allele (OR = 1.51, 95% CI: 1.03–2.20, *p* = 0.032), codominant (OR = 1.54, 95% CI: 1.01–2.35, *p* = 0.044), dominant (OR = 1.57, 95% CI:1.04–2.37, *p* = 0.032), and additive models (OR = 1.54, 95% CI: 1.05–2.26, *p* = 0.029).

**Table 4:** The correlation between *TMOD1* and *PTCSC2* polymorphisms and thyroid carcinoma susceptibility stratified by age and sex

SNP-ID	Model	Genotype	Age ≤ 45		Age > 45		Female		Male	
			**OR (95% CI)**	***p***	**OR (95% CI)**	***p***	**OR (95% CI)**	***p***	**OR (95% CI)**	***p***
rs10982622	Codominant	AG	0.61 (0.41–0.90)	0.014	0.87 (0.60–1.25)	0.443	0.69 (0.50–0.94)	0.019	0.89 (0.54–1.46)	0.631
		GG	1.00		1.00		1.00		1.00	
	Dominant	AA.AG	0.72 (0.50–1.04)	0.084	0.86 (0.62–1.21)	0.390	0.74 (0.55–0.99)	0.043	0.94 (0.59–1.47)	0.773
		GG	1.00		1.00		1.00		1.00	
rs925489	Allele	T	1.00		1.00		1.00		1.00	
		C	1.38 (0.90–2.11)	0.143	1.51 (1.03–2.20)	0.032	1.20 (0.85–1.68)	0.306	2.17 (1.30–3.61)	0.003
	Codominant	CT	1.33 (0.84–2.11)	0.223	1.54 (1.01–2.35)	0.044	1.13 (0.77–1.64)	0.536	2.34 (1.33–4.06)	0.003
		TT	1.00		1.00		1.00		1.00	
	Dominant	CC.CT	1.38 (0.88–2.19)	0.164	1.57 (1.04–2.37)	0.032	1.17 (0.81–1.69)	0.407	2.39 (1.38–4.14)	0.002
		TT	1.00		1.00		1.00		1.00	
	Additive	/	1.43 (0.91–2.22)	0.119	1.54 (1.05–2.26)	0.029	1.20 (0.85–1.69)	0.305	2.39 (1.39–4.13)	0.002
rs965513	Allele	G	1.00		1.00		1.00		1.00	
		A	1.36 (0.88–2.09)	0.165	1.44 (0.98–2.10)	0.059	1.18 (0.84–1.66)	0.347	2.02 (1.21–3.39)	0.007
	Codominant	AG	1.31 (0.82–2.08)	0.260	1.45 (0.96–2.21)	0.083	1.11 (0.76–1.62)	0.600	2.14 (1.23–3.74)	0.007
		GG	1.00		1.00		1.00		1.00	
	Dominant	AA.AG	1.36 (0.86–2.16)	0.193	1.48 (0.98–2.24)	0.061	1.15 (0.79–1.67)	0.460	2.19 (1.26–3.82)	0.006
		GG	1.00		1.00		1.00		1.00	
	Additive	/	1.40 (0.89–2.20)	0.141	1.46 (1.00-2.15)	0.053	1.18 (0.84–1.67)	0.346	2.20 (1.28–3.81)	0.005

SNP: single nucleotide polymorphism; OR: odds ratio; 95% CI: 95% confidence interval

*p* < 0.05 indicates statistical significance. /: indicates additive model

After stratifying by sex, rs10982622 was associated with a significantly reduced risk of thyroid carcinoma for females in the codominant (OR = 0.69, 95% CI: 0.50–0.94, *p* = 0.019) and dominant models (OR = 0.74, 95% CI: 0.55–0.99, *p* = 0.043). For males, rs925489 was associated with an increased risk of thyroid carcinoma under allele (OR = 2.17, 95% CI: 1.30–3.61, *p* = 0.003), codominant (OR = 2.34, 95% CI: 1.33–4.06, *p* = 0.003), dominant (OR = 2.39, 95%CI: 1.38–4.14, *p* = 0.002), and additive models (OR = 2.39, 95% CI: 1.39–4.13, *p* = 0.002). Rs965513 was associated with an increased risk of thyroid carcinoma for males under allele (OR = 2.02, 95% CI: 1.21–3.39, *p* = 0.007), codominant (OR = 2.14, 95% CI: 1.23–3.74, *p* = 0.007), dominant (OR = 2.19, 95% CI: 1.26–3.82, *p* = 0.006), and additive models (OR = 2.20, 95% CI: 1.28–3.81, *p* = 0.005) (Table 4).

### The association of haplotype with thyroid carcinoma risk

**Fig. 1:**
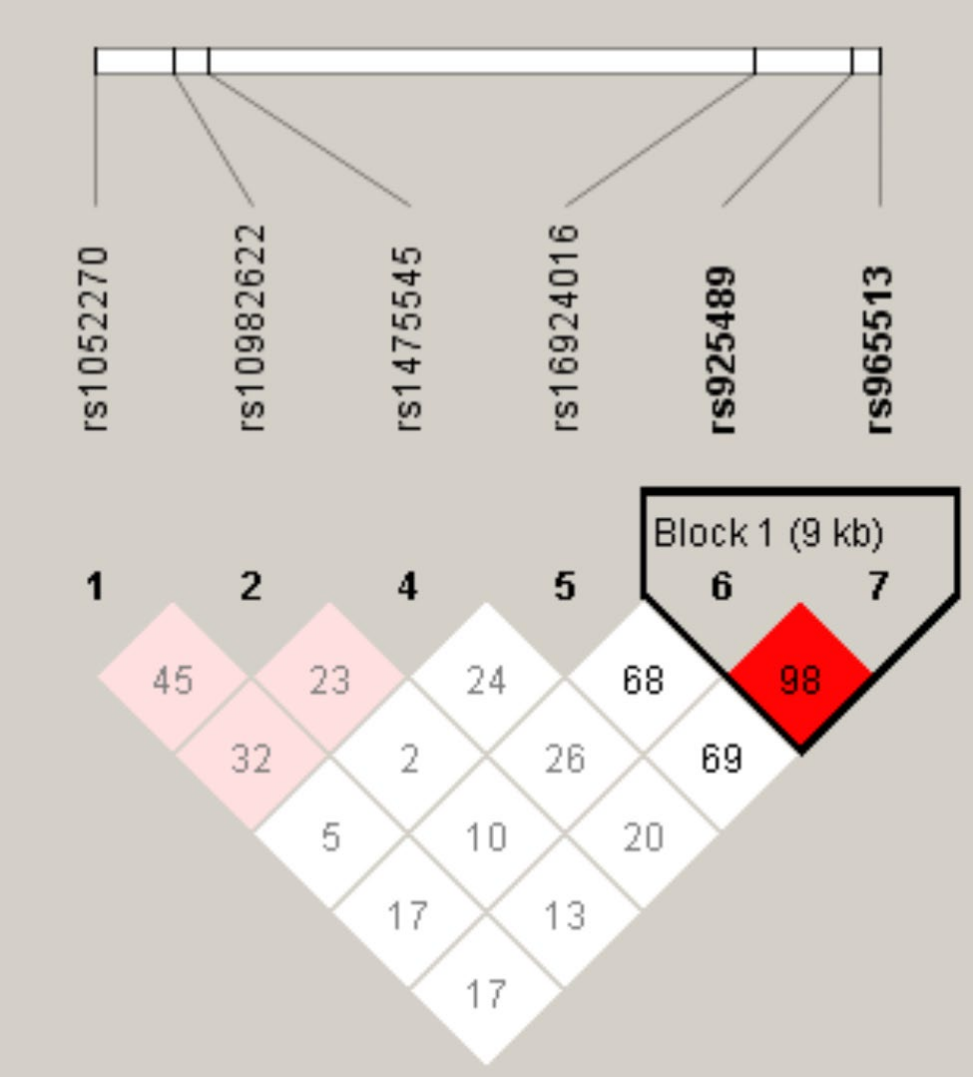
Haplotype block map for SNPs of the *TMOD1* and *PTCSC2* genes. Linkage disequilibrium plots containing six SNPs from *TMOD1* and *PTCSC2*. Red squares display statistically significant associations between a pair of SNPs, as a measured by D’; darker shades of red indicate a higher D’

We further researched the LD and haplotype analyses of the *TMOD1* and *PTCSC2* polymorphisms. The LD plot is represented in Fig. 1 with six SNPs, including rs925489, rs965513, rs1052270, rs10982622, rs1475545, and rs16924016. The results showed that a haplotype block with strong LD between rs925489 and rs965513. We observed the distribution of frequencies for haplotypes in the case group and control groups in Table 5. The haplotype results revealed two remarkable associations of ‘CA/TG’ haplotypes with an increased risk for thyroid cancer (OR = 1.43, 95% CI: 1.07–1.92, *p* = 0.016; OR = 1.47, 95% CI: 1.10–1.97, *p* = 0.009) (Table 5).

**Table 5:** Association between the haplotypes and thyroid carcinoma risk (adjusted for sex and age)

SNP	Haplotype	F_A	F_U	Chi-square	***p*** ^a^	OR (95% CI)	***p*** ^b^
rs925489|rs965513	CA	0.122	0.089	5.598	0.018	1.43 (1.07–1.92)	0.016
	TG	0.127	0.091	6.470	0.011	1.47 (1.10–1.97)	0.009

SNP: Single nucleotide polymorphism; F_A: Frequency in cases; F_U: Frequency in controls; OR: Odds ratio; 95% CI: 95% Confidence interval

***p***^a^ values were calculated using Pearson’s *χ*^*2*^ test. ***p***^b^ values were calculated using the Wald test. ***p*** < 0.05 indicates statistical significance

### Analysis of MDR

As shown in Table 6, the best predictive model for thyroid carcinoma risk was the five-locus model, a combination of rs1052270, rs10982622, rs1475545, rs16924016, and rs925489, with the highest testing accuracy and perfect CVC (testing accuracy = 0.510, CVC = 10/10, *p* < 0.0001). The green and blue connections indicate redundancy or independence among SNPs in Supplementary Fig. 1.

**Table 6:** Summary of SNP–SNP interactions on the risk of thyroid carcinoma analyzed by MDR method

Model	Training Bal. Acc	Testing Bal. Acc	CVC	OR (95% CI)	***p***
rs10982622	0.539	0.505	8/10	1.36 (1.06–1.74)	0.016
rs1052270, rs10982622	0.551	0.506	8/10	1.51 (1.17–1.95)	0.001
rs1052270, rs10982622, rs925489	0.577	0.510	5/10	1.83 (1.42–2.35)	< 0.0001
rs1052270, rs10982622, rs1475545, rs16924016	0.600	0.481	8/10	2.20 (1.70–2.85)	< 0.0001
rs1052270, rs10982622, rs1475545, rs16924016, rs925489^*^	0.628	0.510	10/10	2.73 (2.12–3.52)	< 0.0001
rs1052270, rs10982622, rs1475545, rs16924016, rs925489, rs965513	0.630	0.505	10/10	2.78 (2.16–3.59)	< 0.0001

MDR: multifactor dimensionality reduction; Bal. Acc: balanced accuracy; CVC: cross-validation consistency; OR: odds ratio; 95% CI: 95% confidence interval

***p*** < 0.05 indicates statistical significance. *: the best model in MDR analysis

## Discussion

In this study, we assessed the association of the *TMOD1* (rs1052270, rs10982622, and rs1475545) and *PTCSC2* (rs16924016, rs925489, and rs965513) gene polymorphisms with susceptibility to thyroid carcinoma. Our results showed that rs925489 and rs965513 increased the risk of thyroid carcinoma, while rs10982622 markedly reduced the thyroid carcinoma risk in the Chinese population. These results suggested an association between genetic polymorphisms of *TMOD1* and *PTCSC2* and susceptibility to thyroid carcinoma. Moreover, haplotypes CA and TG (rs925489|rs965513) are risk haplotypes of thyroid carcinoma.

It is still an arduous and long-term task to discover more genetic polymorphisms related to thyroid carcinoma risk. The association between SNPs and thyroid carcinoma susceptibility has been explored in several studies. However, consistent results were not obtained. Some studies discovered that *PTCSC2* plays a key role in the development process of PTC [1, 21, 27]. PTC is a type of thyroid carcinoma. The study first reported the rs965513-related PTC by a GWAS in a European population, and subsequently, the conclusion was replicated in several studies [22, 23, 28–30]. Rs965513 A/G can also increase the risk of thyroid carcinoma in our results, but Kang et al.’s study obtained a different result that the rs965513 was not associated with thyroid carcinoma risk [31]. We speculate that this is due to different types of mutation sites. In addition, our study is the first to report the association between *PTCSC2* rs925489 and thyroid carcinoma risk. Previous studies proved that rs925489 had a strong association with serum TSH [25]. We speculate that rs925489 may affect the occurrence of thyroid carcinoma by regulating TSH levels. Rs10982622 was first found to be a protective factor in thyroid carcinoma because it was not reported previously.

In our results, we found that the association between the polymorphism of *TMOD1* and *PTCSC2* polymorphisms and thyroid carcinoma risk was affected by age and sex. Rs10982622 on *TMOD1* was only first found to protect against thyroid carcinoma in participants in the total analysis and stratification analysis for females. The annual occurrence rate obviously increased by 14.51% for females from 2003 to 2007 [32]. Thyroid carcinoma is the fifth most common new cancer cases in females [33, 34]. The genetic polymorphisms of *TMOD1* may not be a key part of susceptibility to thyroid carcinoma. Studies with larger sample sizes are still needed to seek more statistical findings.

There could be the potential selection bias in our study design. The sample size of our study was relatively small. Studies of the mechanism between *TMOD1* and *PTCSC2* polymorphisms and thyroid carcinoma should be conducted to explore the effect. In summary, our study provides clues for further mechanistic studies, especially in the Chinese Han population. Therefore, we need to validate our findings in larger sample sizes and in different ethnic groups.

## Conclusion

In conclusion, we found that *TMOD1* and *PTCSC2* were correlated with thyroid carcinoma risk in the Chinese Han population. *TMOD1* polymorphism was detected to be markedly decreased, and *PTCSC2* was detected to be markedly increased in thyroid carcinoma risk based on our analysis.

## Electronic supplementary material

Below is the link to the electronic supplementary material.

Supplementary Material 1

## Data Availability

The data used to support the findings of this study are available from the corresponding author upon request.
