# Supplementary figures and images for: Suggestive evidence of the genetic association of TMOD1 and PTCSC2 polymorphisms with thyroid carcinoma in the Chinese Han population
Source: BMC Endocr Disord. 2022 Oct 31;22:263. doi: 10.1186/s12902-022-01177-2 (PMC9620653; doi:10.1186/s12902-022-01177-2)

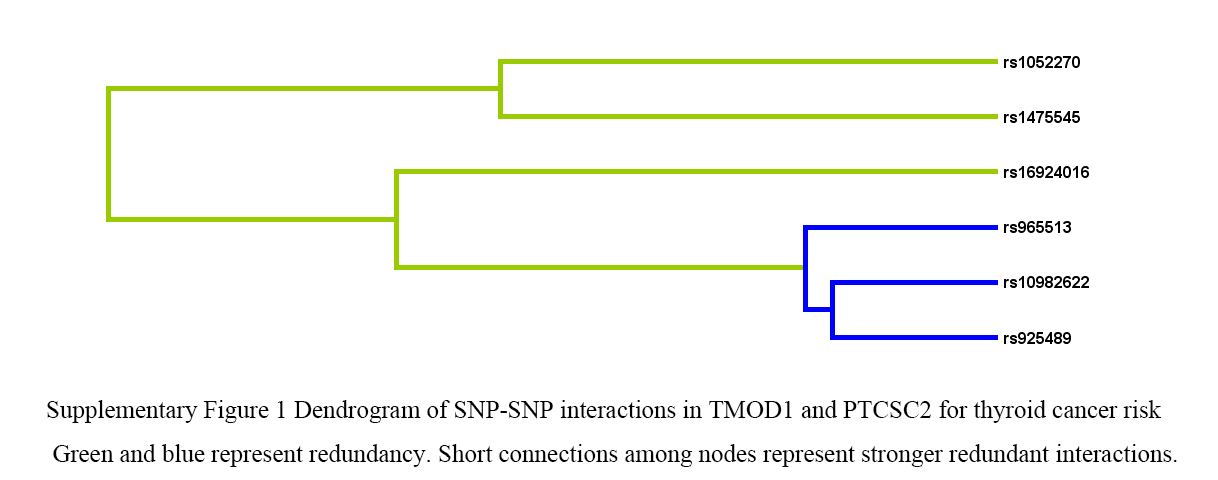

Supplement: Supplementary file 1 — Supplementary Material 1 [file 12902_2022_1177_MOESM1_ESM.png]
